# Evaluation of a Method for the Quantification of Cadmium, Lead, and Zinc in Craft Beers Manufactured in Quito, Ecuador

**DOI:** 10.3390/foods13223664

**Published:** 2024-11-18

**Authors:** Steward Yépez-Basantes, Lenys Fernández, Emerson Maldonado, Wilson Leon-Cueva, Ricardo León-Cueva, Luis Cedeño-Sares, Patricio Espinoza-Montero

**Affiliations:** 1 Escuela de Ciencias Químicas, Pontificia Universidad Católica del Ecuador, Quito 170525, Ecuador; snyepez@puce.edu.ec (S.Y.-B.); pespinoza646@puce.edu.ec (P.E.-M.); 2 Facultad de Ciencias Químicas y de la Salud, Universidad Técnica de Machala, Machala 070222, Ecuador; emaldonado@utmachala.edu.ec (E.M.); wleon@utmachala.edu.ec (W.L.-C.); rleon@utmachala.edu.ec (R.L.-C.); lcedeno@utmachala.edu.ec (L.C.-S.)

**Keywords:** atomic absorption spectroscopy, cadmium, craft beer, lead, standard addition method, heavy metal contamination, Mercosur standards, zinc

## Abstract

The brewing process of craft beer can introduce contamination by heavy metals such as Cd, Pb, and Zn from various sources. Cadmium and lead metals are particularly worrisome because of their harmful effects on human health. This study aimed to evaluate a method for quantifying the levels of Cd, Pb, and Zn in craft beer brands sold in the northern region of the Metropolitan District of Quito, Ecuador, using atomic absorption spectroscopy. For confidentiality, the brands were anonymized as Brands A to I. Standard addition curves were employed for metal quantification, with repeatability assessed via the coefficient of variation (CV%) and accuracy determined by recovery (R%). The Cd content in Brands B–G and I was below the threshold established by the Mercosur Resolution Nº 12/11. Additionally, Brands A and D–H complied with the Ecuadorian INEN 2262 standard for maximum Pb concentration in beer. All samples showed Zn levels substantially below the maximum levels permitted by Ecuadorian regulations. Brands A, B, C, and H exhibited the highest concentrations of Cd and Pb.

## 1. Introduction

Beer, one of the world’s oldest alcoholic beverages, originated over 6000 years ago in the Sumerian region, north of Egypt [1]. While beer production has evolved considerably over time, the risk of contamination by heavy metals such as cadmium (Cd), lead (Pb), and zinc (Zn) remains a concern in modern brewing processes. These metals are particularly worrisome because of their harmful effects on human health. For example, the bioaccumulation of Cd and Pb in the human body can cause renal damage, neurological disturbances, and a higher risk of carcinogenesis, among other issues. Further, while Zn is an essential micronutrient for yeast metabolism and fermentation, it can be toxic at high levels. Other problems related to heavy metal toxicity include anemia, stomach ulcers, nerve damage, muscle numbness or tingling, high blood pressure, and infertility [2].

Heavy metal contamination in beer is a global concern, with multiple studies reporting the presence of metals such as Cd, Pb, and Zn in beers from various regions. For example, research from Brazil, Italy, and the United Kingdom detected Cd and Pb at levels that may pose health risks if consumed regularly [3,4,5]. Sources of contamination in beer include raw materials, water quality, processing equipment, and packaging materials [6,7]. For instance, an American Chemical Society study indicated that Cd and Pb could be introduced into beer through filtration processes using diatomaceous earth [8]. While beer is produced both industrially and artisanally, small-scale craft breweries face distinct challenges in controlling metal contamination compared to larger industrial operations. Smaller production scales, traditional brewing techniques, and potentially less rigorous quality control measures can all contribute to higher levels of metal contamination. For instance, the use of artisanal equipment such as copper vessels or untreated steel tanks can introduce Cd and Pb into the final product [9]. Additionally, unlike the more standardized and regulated supply chains used by industrial breweries, sourcing raw materials from local suppliers can lead to greater variability in metal content.

Given the widespread consumption of beer, the potential exposure to toxic metals has prompted numerous regulatory efforts aimed at controlling heavy metal levels in beverages. Nevertheless, there is a need for careful, continuous monitoring of heavy metal contamination, particularly in craft beer. Evaluating the extent of heavy metal contamination in beer is crucial for ensuring product safety and protecting public health.

Given its sensitivity and operational simplicity, flame atomic absorption spectroscopy (FAAS) is the most commonly used analytical technique for quantifying heavy metals in beer. While more specialized techniques, such as inductively coupled plasma mass spectrometry (ICP-MS) and inductively coupled plasma optical emission spectrometry (ICP-OES), offer lower detection limits (DLs), and they are more expensive and require special sample preparation. The DLs reported for FAAS for Zn, Cd, and Pb are 0.001, 0.001, and 0.008 mg/L, respectively [10], though these may vary slightly depending on the specific equipment used. Despite its higher DLs, FAAS remains an attractive and cost-effective option for the quantification of heavy metals in liquid samples.

As in other parts of the world, beer has a long history in Ecuador. The country’s first brewery was established by Friar Jodoco Rickie and Friar Pedro Glocial in 1566 at the San Francisco Convent in Quito [11,12,13]. Since then, beer has helped shape the history and cultural development of the city. The rise of craft beer in Ecuador began around 2011 in Guayaquil with the introduction of modern brewing recipes and fermentation methods. Today, approximately 150 breweries and microbreweries operate across the country, with around 21 of these located in Quito, as reported by the Craft Beer Association. According to a 2018 article in *El Telégrafo* [14], beer is the preferred alcoholic beverage for the majority of Ecuadorians (56.7%). However, these data contrast with the findings of the National Survey of Income and Expenditures in Urban and Rural Households conducted by the National Institute of Statistics and Censuses [15], which indicated that 79.5% of individuals prefer beer over other alcohol options [16]. These statistics highlight beer’s cultural significance in Ecuador. Nevertheless, despite its popularity, research on the heavy metal content in Ecuadorian craft beer is scarce [7]. Therefore, evaluating the presence of contaminants in these beverages is crucial for a proper risk assessment of craft beer consumption in Quito. This analysis will help determine if these beverages comply with the standards set by the Ecuador Service for Standardization (INEN in Spanish) [16].

To fill this gap, this study aimed to quantify the levels of Cd, Pb, and Zn in nine brands of craft beer manufactured in the Metropolitan District of Quito (MDQ), Ecuador, using FAAS. The objective was to determine whether these beverages complied with the Ecuadorian Technical Norm (NTE in Spanish) INEN 2262 [16] and the Mercosur GMC Resolution Nº 12/11 [17]. As a secondary objective, this study aimed to verify if the conditions outlined in the CODEX STAN 228-2001 standard [18]—450 °C and 1 h and 30 min—were optimal for acid digestion. The study specifically focused on Cd, Pb, and Zn because they are likely to be present in the raw materials and equipment used in Ecuadorian craft breweries and microbreweries [19,20].

## 2. Materials and Methods

### 2.1. Sampling of Craft Beer

A simple random sampling was conducted in three northern zones in the MDQ from nine leading craft beer retailers. The brands were anonymized as Brands A through I. The study focused on red ale, with one brand sampled per week from April 2021 to July 2023. The analysis was performed in duplicate to ensure valid results. Approximately 350 mL of beer was purchased from each location, and samples from different batches of each brand were collected to minimize bias and ensure product representativeness. The variability in heavy metal levels was assessed between different brands, not between different batches. Table 1 provides details of the craft beer samples.

### 2.2. Reagents and Equipment

The following reagents were used: magnesium nitrate (Merck, analytical grade, CAS #: 13446-18-9, Merck KGaA, Darmstadt, Germany), nitric acid (69%, analytical grade, Fisher Chemical, Ottawa, OT, Canada, ACS certificate, CAS# CAS 7697-37-2, PubChem CID: 944), hydrochloric acid (37%, analytical grade, Sigma Aldrich, Steinheim, Germany, Certified ACS, CAS# 10025-69-1, PubChem CID: 24479), and metal-free nitric acid (69%, analytical grade, Fisher Chemical, Ottawa, Canada, ACS certificate, CAS# CAS 7697- 37-2, PubChem CID: 944). Certified standard solutions of Cd, Pb, and Zn were obtained from Merck (Merck KGaA, Darmstadt, Germany, analytical grade). The equipment employed included an ultrasonic bath (Branson 3800, Danbury, CT, USA), hot plate (MTOPO MS100, Quito, Ecuador), muffle furnace (Hanyang Scientific MF-50, Seoul, Republic of Korea), a water purification system (Thermo Scientific MicroPURE UV, Langenselbold, Hungary), and atomic absorption spectrophotometer (PerkinElmer AAnalyst 400, Waltham, MA, USA).

### 2.3. Sample Treatment

Each beer sample was first placed in an ultrasonic bath (Branson 3800) for 20 min. It was then filtered using fine-pore filter paper (185 mm, Macherey Nagel, Lab Unlimited, Carl Stuart Limited, Tallaght Business Park Whitestown, Dublin, Ireland) to remove suspended particulate matter. To prevent the loss of volatile analytes, 0.5 mL of 15% magnesium nitrate (Mg(NO_3_)_2_, Merck) was added to 5 mL aliquots of beer, which were then transferred to porcelain crucibles. These were heated on a hot plate (MTOPO MS100, Thawi Watthana Subdistrict, Thawi Watthana District, Bangkok, Thailand) at 150–180 °C for 20 min to concentrate the samples. After cooling to room temperature, 5 mL of concentrated nitric acid (69%, Merck) was added, and the crucible was heated again to 180 °C to evaporate any remaining nitric acid. The cooled samples were then transferred to a muffle furnace (Hanyang Scientific MF-50, 11 Penns Trail, Suite 300,18940 Newtown, PA, USA) and gradually heated from room temperature to 375 °C at 50 °C/min, maintaining this temperature for 15 min. The temperature was then increased to 450 °C and maintained for 1 h and 20 min. If residual carbon remained, up to two additional calcinations were performed at 450 °C for 15 min each, with the addition of 1 mL of 1:1 HNO_3_. Finally, the ash was dissolved in 2 mL of 8 M hydrochloric acid (37%, analytical grade, Sigma Aldrich, Steinheim, Germany, Certified ACS, CAS# 10025-69-1, PubChem CID: 24479), rinsed with deionized water (Thermo Scientific, MicroPURE UV, TEquipment, 205 Westwood Avenue, Long Branch, NJ, USA), and then diluted to a final volume of 50 mL.

### 2.4. Determination Method of Cd, Pb, and Zn

A modification to the method outlined in the NTE INEN 2330 standard was applied in two sections: sample treatment and analyte determination. During sample treatment, porcelain crucibles were used instead of borosilicate tubes, and after calcination, the ash was dissolved in 2 mL of HCl and diluted to 50 mL with deionized water. For metal quantification, standard addition curves were used, and the analyses were performed in triplicate. Unlike the standard method, direct reading of the treated beer samples was not performed. Instead, DLs and quantification limits (QLs) were calculated using the equations 3*S*/*m* and 10*S*/*m*, respectively, where *m* represents the slope of the calibration curve, and *S* is the standard deviation (SD) of six blank measurements using deionized water. Additionally, an experimental design (Table 2) was implemented to modify the temperature (A) and time (B) during muffle digestion. Table 3 presents the instrumental parameters used, and Table 4 provides the validation parameters according to the acceptable limits set by the Association of Official Agricultural Chemists (AOAC) [13].

### 2.5. Quantification of Cd, Pb, and Zn in Beer

To quantify Cd, Pb, and Zn in beer, standard addition plots were prepared in duplicate. Standard solutions were derived from a certified 1000 mg L^−^^1^ standard for each metal in 0.5 M HNO_3_ (Merck). The concentrations for the standard solutions were set at 10 mg/L, 20 mg/L, and 10 mg/L for Cd, Pb, and Zn, respectively. Nine equidistant calibration points were established, with consistent increments of 0.5 between each pair, including the reagent blank. The ranges for the standard addition plots were as follows: 0.05–0.40 mg/L for Cd; 0.5–4.0 mg/L for Pb; and 0.10–0.45 mg/L for Zn. Each standard plot was prepared by combining 2 mL of the treated beer sample with a known volume of standard solution (mL), then diluting to a final volume of 50 mL with 0.1 M metal-free HNO_3_ (Merck). Measurements were obtained using a PerkinElmer AAnalyst 400 atomic absorption spectrophotometer (SpectraLab Scientific Inc., 38 McPherson St., Markham, ON, Canada, L3R 3V6).

### 2.6. Calculations and Data Treatment

Data were processed using Excel software 2019, which facilitated calculations for linear regression, linear ranges, mean values, SD, coefficient of variation (CV%), recovery (R%), sensitivity, method DLs, and method QLs. Repeatability was evaluated through CV%, calculated using the equation CV = SD/$\bar{X}$, while accuracy was assessed by R%, calculated using the equation


$$R=\frac{\left[\mathrm{Measured}\;\mathrm{concentration}-\mathrm{Analyte}\;\mathrm{concentration}\;\mathrm{in}\;\mathrm{reading}\right]}{\mathrm{Concentration}\;\mathrm{of}\;\mathrm{the}\;\mathrm{added}\;\mathrm{standard}}.$$

The analyte concentration in the readings was determined using the formula *b*/*m*. Validation parameters were evaluated based on acceptable limits established by the AOAC for validating chemical methods in laboratories for dietary supplements and botanicals.

Descriptive statistics, including mean, SD, range, and R%, were calculated using Microsoft Excel 2019 and BioEstat 5.3. The normality of data distribution was assessed using the Shapiro–Wilk test. As the data did not follow a normal distribution, non-parametric tests were performed. The Mann–Whitney U test was used to compare significant differences between the nine brands study from one site of Quito, while the Kruskal–Wallis test was employed to determine significant differences in metal content of each beer brand individually.

## 3. Results

**Figure 1:**
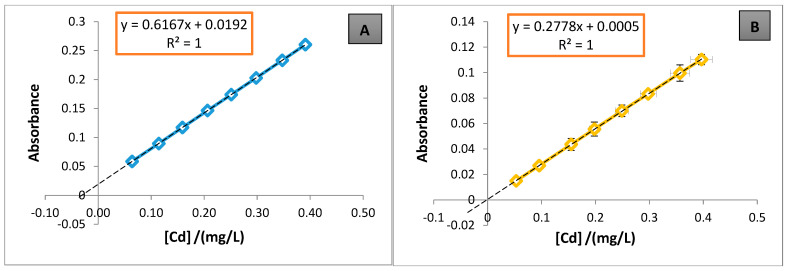
Standard addition calibration plots for Cd quantification; (**A**) corresponds to Plot 1-A and (**B**) corresponds to Plot 2-A from Table 5.

**Figure 2:**
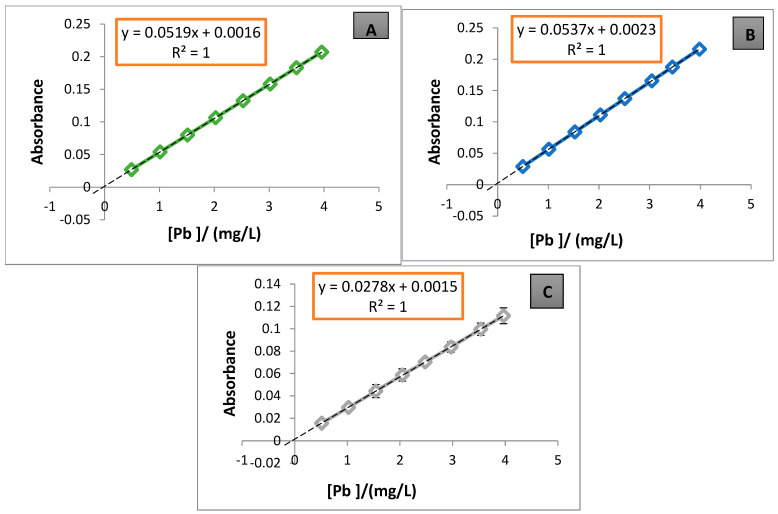
Standard addition calibration plots for Pb quantification; (**A**) corresponds to Plot 3-A, (**B**) corresponds to Plot 4-A, and (**C**) corresponds to Plot 5-A from Table 5.

**Figure 3:**
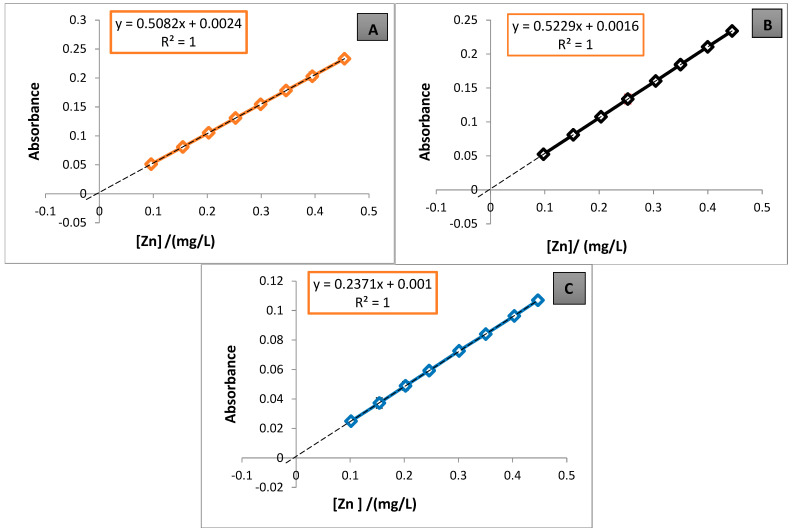
Standard addition calibration plots for Zn quantification (**A**) corresponds to Plot 6-A, (**B**) corresponds to Plot 7-A, and (**C**) corresponds to Plot 8-A from Table 5.

Table 5 shows the statistical validation parameters for the method and the duration of the analysis in weeks. Each parameter represents the average of three standard addition plots, with Brand A used exclusively used for the validation process. The calibration plots for the quantification of each metal in the samples are shown in Figure 1, Figure 2 and Figure 3.

Table 6 presents the average Cd concentration in nine craft beer brands, along with the average SD, CV%, and R%. Additionally, compliance with the Mercosur Resolution Nº 12/11 [17] was evaluated, considering its maximum permissible limit for Cd. The Kruskall–Wallis test revealed significant differences in Cd levels between the brands (*p* < 0.01), and the Wilcoxon test confirmed that the Cd content in Brands A, H, and I significantly differed from that in the other brands. This variation may be attributed to differences in the matrix composition of the particular samples. Craft beers often exhibit diverse chemical compositions due to the unique ingredients and brewing processes used by each brand. These components can affect the efficiency of Cd digestion or extraction. Brands A, H, and I may contain certain organic compounds or minerals that interfere with Cd quantification, which may explain this behavior.

Table 7 shows the average Pb concentration in nine craft beer brands, as well as the average SD, CV%, and R% and the compliance with NTE INEN 2262 [16]. Similar to the results for Cd (Table 6), the statistically significant differences in Pb content were likely due to variations in the matrix composition of these particular samples, as the differences coincide with the same Brands A, H, and I in the Cd analysis.

Table 8 presents the average Zn concentration in eight craft beer brands and the average SD, CV%, and R%, as well as the compliance with NTE INEN 2262 [16]. The Kruskal–Wallis test revealed no significant differences in Zn levels between the brands (*p* < 0.01). In contrast to Cd and Pb, where significant variations were observed across brands, Zn appeared to be more uniformly distributed. This could be because Zn is an essential element in the brewing process, serving as a micronutrient for yeast and other ingredients; therefore, it is subject to stricter regulation. As a result, Zn levels were likely more consistent across all brands. On the other hand, Cd and Pb contamination may originate from various sources, such as raw materials, packaging, or processing equipment, leading to greater variability in their concentrations across brands. This variability may be due to less stringent control over these metals compared to Zn within the brewing industry.

## 4. Discussion

The results of this study highlight the importance of optimizing the dry digestion process for accurate metal quantification in craft beer samples, particularly regarding the effects of varying temperature and time. To evaluate these conditions, an experimental design was implemented, as shown in Table 2, where different combinations of high and low temperature (A) and time (B) were tested. This experiment was performed to verify whether the conditions of 450 °C and 1 h and 30 min, as outlined in the INEN 2330 standard [18], were optimal for the second dry digestion process in the muffle furnace. At 400 °C, the calcination of the sample remained incomplete, with visible carbon residue in the crucible, even after extending the digestion time from 1 h to 1 h and 30 min. In contrast, increasing the temperature to 450 °C significantly reduced the carbon residue, indicating a more complete calcination process. This finding suggests that while time adjustments had minimal impact on sample treatment, temperature was a critical factor for ensuring complete digestion. The conditions of 450 °C and 1 h and 30 min were confirmed as the most suitable for achieving optimal acid digestion, as recommended by the INEN standard [18]. Although previous studies have reported that dry ash mineralization is an inefficient digestion method, citing potential contamination risks, volatile element loss, and lower recoveries compared to other digestion procedures [8], the accuracy values in this study, as shown in Table 2, were favorable. This success can be attributed to the pre-concentration step included in the sample treatment, which minimized the potential for significant analyte loss.

To buttress the results of this study and due to the lack of the use of CRM as a quality assurance method, strict measures were put in place to ensure that accurate and precise results were obtained. These controls involved working with fortified samples, repeated analysis, and taking all necessary steps to achieve the required data quality to detect Cd, Pb, and Zn in craft beer samples. Despite the fact that CRM offers traceability connected to international standards, the internal control currently implemented offers sufficient reliability for the objectives of this study, as well as generating coherent and replicable results within the framework of this investigation. The method validation results for the quantification of each metal using Brand A are presented in Table 5. In the case of Cd, two validations were performed; over weeks 1–3, an average Cd concentration of 0.7783 mg/L was obtained, which exceeded the maximum permissible limit. The SD of 0.0029% and CV% of 2.02% were both within acceptable validation limits. R% was 84.87%, indicating efficient analyte recovery. The standard addition curve in Figure 1A shows strong linearity, with a correlation coefficient (*R*^2^) of 1 and low signal error (SD), confirming the reliability of the data for these weeks. In weeks 4–6, a lower Cd concentration of 0.0451 mg/L was observed, falling below the QL, even though the same brand and beer type were used. The CV% increased to 8.01%, exceeding the acceptable threshold of 6.00%. In Figure 1B, this higher CV% is observed for calibration points 3, 4, and 7 in Plot 2-A. However, despite a high R% of 99.26%, these results were considered not acceptable for validation.

For Pb quantification, three validations were performed to ensure method reliability. In weeks 7–9, the Pb content was 0.7704 mg/L, which exceeded the permissible limit. This value was below the method’s QL, with an SD% of 0.0008% and a CV% of 0.68%, both acceptable for validation. Therefore, the value obtained from the standard addition plot can be considered acceptable or referential given the very low analyte concentration [6]. R% was 98.07%, and Figure 2A shows optimal linearity, with *R*^2^ = 1 and low signal error (SD). These results were thus deemed reliable. In weeks 10–12, the Pb concentration rose to 1.071 mg/L, again exceeding the permissible limit. The SD% was 0.0036% and the CV% 3.46%, both within acceptable validation limits. The standard addition curve in Figure 2B demonstrates optimal linearity, with *R*^2^ = 1 and acceptable signal error (SD) at calibration points 5 and 7 in Plot 4-A. Thus, the values for weeks 10–12 are considered reliable for validation. In weeks 13–15, the Pb content peaked at 1.350 mg/L, exceeding the regulation’s permissible limit, with an SD of 0.0045% and a CV% of 8.67%. The latter value was outside the acceptable validation criterion, but an R% of 97.21% was achieved. Further, Figure 2C shows adequate linearity, (*R*^2^ = 1), though with higher signal error (SD) at several points compared to Curve A, Figure 1A. These results were considered reliable, despite the increased variability.

For Zn, three validations were performed over weeks 16–18, 19–21, and 22–24, yielding concentrations of 0.1182, 0.0765, and 0.1053 mg/L, respectively. In terms of SD%, CV%, and accuracy, these results were the best out of all the analyses. The second value was below the QL, but all remained below the threshold set by the INEN 2262 standard [16]. The SD% did not exceed 3.00%, and the CV% remained below the 6.00% acceptance parameters for validation. Figure 3A–C show the standard addition curves for weeks 16–24, displaying optimal linearity (*R*^2^ = 1) and acceptable signal error (SD). After analysis, these results were considered reliable.

In the extrapolations of the standard addition plots (Figure 1, Figure 2 and Figure 3) to the negative axis of the concentration (abscissa), most of the plots pass very close to the origin, except for Figure 1A, where a noticeable shift is observed. This shift may be due to the low analyte concentrations in the matrix [7], which can affect the accuracy of the linear extrapolation. This study utilized standard addition plots to quantify Cd, Pb, and Zn, specifically to mitigate these effects. Consequently, when analyte levels are below 0.1 mg/L, shifts in the standard addition plots on the absorbance axis become less detectable [6]. The method’s DL and QL were 0.0546 and 0.1821 mg/L for Cd, 0.2543 and 0.8476 mg/L for Pb, and 0.0291 and 0.0969 mg/L for Zn, respectively. These values suggest that the method employed—acid digestion on a hotplate, followed by dry digestion in a muffle furnace—using standard addition plots is sensitive for concentrations above 0.18 mg/L for Cd, 0.85 mg/L for Pb, and 0.10 mg/L for Zn. Notably, Pb showed lower sensitivity (S = 0.0847 mg/L), with a relatively high DL, indicating that the FAAS technique is less effective for detecting very low Pb concentrations, as previously reported [6].

For the quantification of Cd, Pb, and Zn in various craft beer brands, duplicate analyses were conducted. Table 6 summarizes the Cd content in Brands A to I, as well as other metrics. For Brands B, C, D, E, F, G, and I, Cd the concentrations were 0.0759, 0.0126, 0.0668, 0.1015, 0.1147, 0.0873, and 0.1700 mg/L, respectively. Most brands exhibited a CV% below the 6.00% limit established by the AOAC, except for Brand I, which had a CV% of 6.82%. Additionally, R% was high across all brands. Although most brands had Cd concentrations above the maximum permissible limit, compliance was assessed using the QL. For example, Brand E had a Cd concentration of 0.1015 mg/L, which exceeded the regulatory threshold of 0.0200 mg/L, but was below the QL of 0.1821 mg/L for the applied method. Therefore, Brand E was deemed compliant, with no detectable Cd, and the result is considered reliable. Conversely, Brands A and H had Cd concentrations of 0.7783 and 0.3029 mg/L, respectively, exceeding Mercosur’s permitted maximum level. While both brands exhibited a CV% below 5.00% and high R% values, Brand A’s R% was very close to the lower acceptable limit of 80.00%, with a value of 84.87%. It is important to note that the Cd content in Brand A was excessively high, surpassing the tolerable intake level of 2.5 µg/kg of body weight [2]. The elevated Cd concentrations in these samples surpassed those reported by López-Balladares et al. [7], which is concerning. Consuming large amounts of Cd-contaminated beer, especially at elevated levels, could lead to serious health consequences, including renal necrosis, hypertension, liver damage, and lung cancer [7]. Cd, as a cumulative toxin, poses a substantial risk to consumers, particularly when consumed regularly. Chronic Cd exposure predominantly impacts the kidneys, resulting in potential chronic kidney disease [21]. Furthermore, the International Agency for Research on Cancer has classified Cd as a carcinogen, meaning that prolonged exposure increases the risk of cancer. Cd has also been linked to bone demineralization and other skeletal disorders [22]. Thus, even modest amounts of Cd in beer can present severe public health risks with regular consumption.

Table 7 presents the Pb content in Brands A to I, as well as other values. The Pb concentrations for Brands A, D, E, F, G, and H were 0.7704, 0.4022, 0.6812, 0.7044, 0.5378, and 0.8227 mg/L, respectively. Most of these beers exhibited a CV% below the acceptable limit of 6.00%, except for Brands H and I, which had a CV% of 9.40%. R% was high across all brands. All the evaluated beers exceeded the maximum permissible Pb concentration set by INEN 2262 [16]; however, the QL criterion was used to evaluate their compliance. Brand I exhibited a Pb content of 1.015 mg/L, with a CV% of 8.91%. Brands B and C had Pb concentrations of 0.122 and 0.9490 mg/L, respectively, both surpassing the maximum allowed by the INEN 2262 standard [16]. Both brands had a CV% below 4.00% and similar R%. The Pb content in both brands exceeded the recommended daily intake of 0.3 and 0.5 mg Pb/day, as well as the WHO’s tolerable weekly intake of 25 µg/kg of body weight [23,24]. Compared to previous studies, the Pb concentrations found in the beers in the current study were higher than those reported by Senila et al. [25].

Excessive Pb bioaccumulation can result in kidney and liver damage, impaired vitamin D metabolism, harm to the central nervous system, and anemia, among other issues [7]. It can also cause neurotoxicity, particularly affecting cognitive development in children and leading to neurological disorders in adults. Chronic Pb exposure also affects the cardiovascular system, increasing the risk of hypertension and heart disease. Additionally, Pb accumulates in bones, potentially causing long-term health effects even after exposure ends [26,27].The consumption of beer with Pb contamination, even at low levels, represents a significant public health concern. As Pb is not highly sensitive to detection using FAAS, the instrument’s QL for Pb is relatively high at 0.3 mg L^−^^1^ [6]. In the current study, the measured concentrations were no lower than 0.4 mg/L (Table 7). To achieve more sensitive and cost-effective Pb quantification, which would reduce the need to use certified standards, electrochemical techniques, such as differential pulse anodic stripping voltammetry, could be considered for quantifying Pb^2^^+^ in the µg/L range [7,28].

Regarding Zn, Table 8 presents the Zn content in Brands A to I, as well as SD%, CV%, R%, and compliance with the INEN 2262 standard [16]. Zn concentrations for Brands A and C–I were 0.0765, 0.0935, 0.1637, 0.1055, 0.0290, 0.0058, 0.0212, and 0.0091 mg/L, respectively. All brands had a CV% below 4.00%, indicating good repeatability, with R% values that were not lower than 97.37%. All brands exhibited Zn concentrations below the maximum permissible value of 1.000 mg/L set by the current regulation. The highest Zn content was observed in Brand D. Compared to the findings by Senila et al. [25], the Zn concentrations in the current study were higher. Nevertheless, the Zn content in these brands did not pose a health risk to consumers, as Zn is an essential metal for various biological functions, including immune response, enzyme activity, and DNA and protein synthesis. The recommended daily intake of Zn is 11 mg for adult men and 8 mg for adult women [29]. Zn toxicity, typically occurring at intakes between 4 and 8 g per day, can cause nausea, fever, and vomiting [30]. Intakes above this level may impair immune function and reduce the levels of good cholesterol (HDL).

The permissible limits of Cd, Pb, and Zn used in this study were based on the Ecuadorian INEN 2262 standard [16] and the Mercosur Resolution GMC Nº 12/11 [17], which closely align with international regulations. For example, the European Commission Regulation (EC) No 1881/2006 establishes a maximum limit of 0.02 mg/L for Cd and 0.10 mg/L for Pb in beverages, while the US Food and Drug Administration (FDA) has a stricter limit of 0.005 mg/L for both Cd and Pb in drinking water [24]. Zn regulation is less centralized, with the FDA setting a limit of 5 mg/L in food products but no specific limit for alcoholic beverages. The thresholds used in the current study were within these internationally accepted ranges, ensuring that our findings are globally relevant and comparable.

## 5. Conclusions

Cd, Pb, and Zn were successfully quantified in nine craft beer brands sold in the northern region of the MDQ using FAAS, based on a representative random sample. The method, adapted from the NTE INEN 2330 standard with minor modifications, was validated for all three analytes, achieving optimal percentages of SD, repeatability, and accuracy in most cases, and meeting the acceptable validation limits. For Cd, Brands B–G and I complied with the Mercosur Resolution Nº 12/11 standard for fermented alcoholic beverages, while Brands A and H exceeded the regulatory limit. For Pb, Brands A and D–I complied with the INEN 2262 standard for the maximum permissible Pb concentration in beer, while Brands B and C had concentrations far above the established limit. Finally, in the case of Zn, all brands showed levels far lower than the maximum allowed by INEN 2262, with Brand D showing the highest Zn content. The applied method of acid digestion on a heating plate, followed by dry digestion in a muffle furnace, proved highly effective for Zn quantification.

The high levels of heavy metal contamination observed in Brands A, B, C, and H, as well as the lower concentrations in the other brands, could be attributed to various stages of the craft beer manufacturing process, including raw materials, brewing equipment such as filtration systems, or cross-contamination. However, pinpointing the exact stage where contamination occurred was not feasible based on the current data. A comprehensive evaluation of the brewing process in each brewery and microbrewery within the MDQ would be required to determine the precise sources of contamination.

## Figures and Tables

**Table 1:** Identification of craft beer samples.

Brand Code	Origin ^a^	Sale Location	Specific Ingredients	Style and ABV ^b^
RED-A	Ecuador	Avenue Foch	Caramel barley malt, fruity and citrus hops	India pale ale-ALT, 5.0%
RED-B	Ecuador	Avenue 12 de Octubre	Caramel barley malt, honey, floral and citrus hops	Red ale, 7.5%
BLON-C	Ecuador	Avenue Foch	Barley malt, caramelized malt, banana peel	Blonde ale, 4.5%
RED-D	Ecuador	Avenue Oswaldo Guayasamin	Caramel barley malt, roasted barley, fruity hops	Red ale, 6.0%
RED-E	Ecuador	Avenue 6 de Diciembre	Caramel barley malt, blackberry, fruity hops	Red ale, 7.0%
RED-F	Ecuador	Avenue Whymper	Caramelized malt, roasted barley, floral and citrus hops	Red ale, 5.3%
RED-G	Ecuador	Avenue Andalucia	Caramel and toffee-flavored malt, floral and citrus hops	Red ale, 6.0%
RED-H	Ecuador	Avenue Foch	Caramel barley malt, cereal malt	Red ale, 5.3%
RED-I	Ecuador	Avenue Whymper	Caramelized malt, roasted barley, citrus hops	Red ale, 5.0%

^a^ In the city of Quito. ^b^ All beers were red ales except for BLON-C, which was a blonde ale.

**Table 2:** Variables for an experimental 2^k^ factorial design.

Temperature (A)/°C	Time (B)/h
400	1
450	1
400	1:30
450	1:30

**Table 3:** Instrumental parameters for determining Cd, Pb, and Zn levels in craft beer.

Parameter	
Flame type	Air–Acetylene
Gas flow (L/min)	10/2.5
**Cd**	
λ (nm)	228.80
Slit width	2.7/1.35
Current (mA)	210
Energy lamp	38
Lamp type	Electrode discharge
Instrumental detection limit (mg/L)	0.003
Instrumental quantification limit (mg/L)	0.01
**Pb**	
λ (nm)	217.00
Slit	2.7/1.35
Current (mA)	450
Energy lamp	54
Lamp type	Electrode discharge
Instrumental detection limit (mg/L)	0.1
Instrumental quantification limit (mg/L)	0.3
**Zn**	
λ (nm)	213.86
Slit	2.7/1.8
Current (mA)	15
Energy lamp	46
Lamp type	Hollow cathode
Instrumental detection limit (mg/L)	0.017
Instrumental quantification limit (mg/L)	0.05

**Table 4:** Acceptable limits of validation parameters for quantifying Cd, Pb, and Zn in craft beer according to AOAC standards.

Parameter	Concentration (µg/g)	Acceptable Limit (AOAC)
Accuracy (R%)	10	80–120
Repeatability (CV%)	10	6
Standard deviation (SD%)	10	3
Correlation coefficient (*R*^2^)	----------	0.995

Note: AOAC: Association of Official Agricultural Chemists.

**Table 5:** Validation parameters of Brand A for each metal (*n* = 3).

Cd									
	Sample	Linear Interval (mg/L)	Concentration in Beer Sample (mg/L) *	SD (%)	CV (%)	R (%)	Sensitivity (mg/L)	Detection Limit (mg/L)	Quantification Limit (mg/L)
Weeks 1, 2, 3									
	Plot 1-A	0.18–0.40	0.7783	0.2936	2.02	84.87	0.0071	0.0546	0.1821
Weeks 4, 5, 6									
	Plot 2-A	0.18–0.40	0.0451	0.3975	8.01	99.26	0.0159	0.0546	0.1821
**Pb**									
Weeks 7, 8, 9									
	Plot 3-A	0.85–4.00	0.7704	0.0803	0.68	98.07	0.0847	0.2543	0.8476
Weeks 10, 11, 12									
	Plot 4-A	0.85–4.00	1.071	0.3647	3.46	97.53	0.0819	0.2543	0.8476
Weeks 13, 14, 15									
	Plot 5-A	0.85–4.00	1.350	0.4535	8.67	97.21	0.1584	0.2543	0.8476
**Zn**									
Weeks 16, 17, 18									
	Plot 6-A	0.10–0.45	0.1182	0.4564	3.59	97.78	0.0087	0.0291	0.0969
Weeks 19, 20, 21									
	Plot 7-A	0.10–0.45	0.0765	0.2917	2.30	98.80	0.0084	0.0291	0.0969
Weeks 22, 23, 24									
	Plot 8-A	0.10–0.45	0.1053	0.2270	4.30	98.51	0.0185	0.0291	0.0969

* Metal concentration found in the samples is highlighted in red.

**Table 6:** Total Cd in craft beer samples.

Beer Brand	Cd (mg/L)	SD (%)	CV (%)	R (%)	Mercosur Resolution Nº 12/11 (mg/L)	Compliant with Relevant Regulation?
A	0.7783	0.2936	2.02	84.87	0.0200	No
B	0.0759	0.6276	4.62	98.52	0.0200	Yes
C	0.0126	0.4349	4.54	98.81	0.0200	Yes
D	0.0668	0.1679	3.70	98.97	0.0200	Yes
E	0.1015	0.2298	4.44	98.12	0.0200	Yes
F	0.1147	0.3536	5.43	95.71	0.0200	Yes
G	0.0873	0.1679	4.00	97.31	0.0200	Yes
H	0.3029	0.1945	4.05	93.84	0.0200	No
I	0.1700	0.3447	6.82	97.77	0.0200	Yes

Note. The metal concentration found in the samples is highlighted in red. SD: standard deviation; CV: coefficient of variation; R: recovery.

**Table 7:** Total Pb in craft beer samples.

Beer Brand	Pb (mg/L)	SD (%)	CV (%)	R (%)	NTE INEN 2262 (mg/L)	Compliant with Relevant Regulation?
A	0.7704	0.0803	0.68	98.07	0.1000	Yes
B	0.1220	0.3297	3.35	97.42	0.1000	No
C	0.9490	0.2254	2.53	97.90	0.1000	No
D	0.4022	0.1149	2.10	98.54	0.1000	Yes
E	0.6812	0.1989	2.46	97.68	0.1000	Yes
F	0.7044	0.1591	2.37	98.36	0.1000	Yes
G	0.5378	0.2210	2.52	99.33	0.1000	Yes
H	0.8227	0.4773	9.40	98.45	0.1000	Yes
I	1.0150	0.6541	8.91	97.67	0.1000	Yes

Note. The metal concentration found in the samples is highlighted in red. SD: standard deviation; CV: coefficient of variation; R: recovery.

**Table 8:** Total Zn in craft beer samples.

Beer Brand	Zn (mg/L)	SD (%)	CV (%)	R (%)	NTE INEN 2262 (mg/L)	Compliant with Relevant Regulation?
A	0.0765	0.2917	2.30	98.80	1.000	Yes
B	NA	NA	NA	NA	1.000	NA
C	0.0935	0.2934	2.07	98.45	1.000	Yes
D	0.1637	0.1149	1.87	97.37	1.000	Yes
E	0.1055	0.2298	3.37	98.35	1.000	Yes
F	0.0290	0.0707	1.30	99.98	1.000	Yes
G	0.0058	0.0707	1.07	99.84	1.000	Yes
H	0.0212	0.1945	3.23	99.19	1.000	Yes
I	0.0091	0.1679	3.10	99.63	1.000	Yes

Note. The metal concentration found in the samples is highlighted in red. SD: standard deviation; CV: coefficient of variation; R: recovery. NA = not applicable; calibration points outside the linearity of the plot.

## Data Availability

The raw data supporting the conclusions of this article will be made available by the authors on request.
